# Prevalence of chronic kidney disease in the Netherlands and its cardiovascular and renal complications

**DOI:** 10.1186/s12882-023-03384-y

**Published:** 2023-11-13

**Authors:** Marc G. Vervloet, Hilda JI de Jong, Jan Pander, Jetty A. Overbeek

**Affiliations:** 1 Nephrology Department, Amsterdam UMC, Amsterdam, The Netherlands; 2 PHARMO Institute for Drug Outcomes Research, Utrecht, The Netherlands; 3 Evidence Generation Lead, AstraZeneca BV, The Hague, The Netherlands

**Keywords:** Cardiorenal Complications, Chronic Kidney Disease, Diabetes Mellitus, Heart Failure, Prevalence

## Abstract

**Background:**

Knowledge on prevalence, comorbidities and consequences of chronic kidney disease (CKD) is mandatory to estimate the potential of cardiovascular risk management on a population level. We studied the prevalence of CKD with or without type 2 diabetes mellitus (T2D) and/or heart failure and its cardiorenal complications in The Netherlands.

**Methods:**

A descriptive cross-sectional and longitudinal cohort study was performed, using data from the Dutch PHARMO Data Network. Prevalence of CKD at a single time point was determined by a recorded diagnosis or by ≥ 2 estimated glomerular filtration rate (eGFR) measurements and urine albumin/creatinine ratio (UACR) that define CKD. A representative group of adults with CKD was included in a longitudinal analysis to study cardiorenal complications. Those were followed until first complication, end of study or death, whichever occurred first.

**Results:**

The prevalence of CKD was 8.9% in a representative population of 2,187,962 adult Dutch individuals. The average age of persons with CKD was 72 years, 57% were female, 19.9% had T2D, 7.7% heart failure, and 3.0% both T2D and heart failure. In the longitudinal analysis, cerebrovascular events (11/1,000 person-years), hospitalizations for heart failure (10/1,000 person-years), myocardial infarction (5.5/1,000 person-years), and hospitalization for CKD (6.2/1,000 person-years) were the most common first cardiorenal complications. People with CKD with T2D and/or heart failure generally had higher rates of cardiovascular or renal complications or mortality than people with CKD without these comorbidities.

**Conclusion:**

The prevalence of CKD in The Netherlands is 8.9%. People with T2D or heart failure, or both, in addition to CKD, had numerically higher mortality and cardiorenal complication rates than people without these comorbidities. Optimizing up-to-date cardiovascular risk management in these high-risk individuals may provide health benefits.

**Supplementary Information:**

The online version contains supplementary material available at 10.1186/s12882-023-03384-y.

## Background

Chronic kidney disease (CKD) is defined as a glomerular filtration rate (GFR) < 60 ml/min/1.73 m^2^ for a period of at least three months or albuminuria of at least 30 mg/g creatinine or 3 mg/mmol. A widespread perception is that the predominant health risk of CKD is progression to end-stage kidney disease (ESKD). However, this occurs in only a small number of patients [1]. The dominating health risks for people with CKD are hospitalizations, cardiovascular (CV) complications, CV death, and all-cause mortality before reaching ESKD [2, 3]. The risks of these complications for people with CKD are similar to those who have diabetes mellitus with heart failure. Many people with CKD in The Netherlands will also have diabetes mellitus and/or heart failure, but their proportion is not well-established. Although the burden of CKD increases globally, there is limited information on the overall prevalence of CKD in the Netherlands.

A previous study from The Netherlands (PREVEND) reported a prevalence of 10.4% [4]. This study was confined to one region and was intentionally oversampled for people with albuminuria. In addition, this study actively screened for CKD among participants with no previous diagnosis of kidney disease. Another study reported a prevalence of 5.1% but essentially captured data from the general practices only [5].

Importantly, it is also unknown whether the simultaneous existence of CKD, diabetes mellitus and heart failure further increases the risk of complications. Detailed knowledge of the magnitude of potential additional risks of these comorbidities can answer the question of which patient categories could benefit most by optimizing current cardiovascular risk management or implementing the most recent treatment strategies in primary practitioner or hospital-based health care.

This study therefore investigated the prevalence of CKD in The Netherlands in a large contemporary representative cohort using several linked databases capturing known CKD cases from primary care and hospital-based records. Also, the study investigated the risk of cardiorenal complications in a representative subset of this population and whether diabetes mellitus and/or heart failure, in addition to CKD, increases these risks.

## Methods

### Data collection

The data for this study were obtained from the PHARMO Data Network. This population-based network combines data from different primary and secondary healthcare settings, including data from the general practitioner (GP), in-hospital and outpatient settings, and pharmacy data in The Netherlands [6]. These data sources are linked at the patient level through validated algorithms.

The GP data include medical records – reported diagnosis and symptoms (based on the International Classification of Primary Care [ICPC] coding) [7], laboratory results, referrals to hospital care, and medication prescriptions – collectively shown to be representative for the Dutch population [8]. The prescription records include information on the product type, prescription date, dose, use, quantity, and route of administration. The hospital data contain admission data from the Dutch Hospital Data foundation of more than 80% of all Dutch hospitals, with balanced representation of the different types of hospitals in The Netherlands (e.g. academic, teaching and peripheral hospitals). Information on admission and discharge date, discharge diagnoses (based on WHO ICD coding [9]), and procedures (based on NZa (*Nederlandse Zorgautoriteit* [Dutch Healthcare Authority]) declaration codes and Dutch Classification of Procedures [10]) were available. The outpatient pharmacy data comprises healthcare products the GP or medical specialist prescribes.

### Study population

For the objectives of this study, the following cohorts were selected:

1. **Prevalence cohort**: To study the most recently available prevalence of CKD, all people with GP data were selected on 31 December 2019. This date was chosen due to the change in care during the coronavirus disease-19 (COVID-19) pandemic (in 2020). No other analyses were based on this cohort.
2. **Outcomes cohort**: To determine the renal and cardiovascular complications over two years, people with GP data AND the possible availability of hospital data (because they live in the catchment area of the affiliated hospitals), were selected. The index date for this analysis was 31 December 2017, allowing two years follow-up until 31 December 2019. Due to the linkage of these databases, the need for geographical overlap, and the required availability of longitudinal data, the number of patients in this cohort was lower than in the prevalence cohort.

### Privacy statement

Because only anonymized data from the PHARMO Data Network was used for this study, the study is not subject to ethics review according to the Medical Research Involving Human Subjects Act (WMO). The institutional review board of STIZON, Utrecht, Netherlands approved the study. The institutional review board of STIZON consists of representatives of all different healthcare providers and a separate privacy expert, including the GDPR data protection officer of STIZON. All studies require permission of the Compliance Committee and its decisions are based on the applicable Dutch and European legislations, including the Medical Treatment Contracts Act (WGBO).

### Study design

This is a descriptive cohort study with a cross-sectional design to describe the prevalence of CKD (cohort 1) and a longitudinal design to describe the event rates of cardiorenal complications (cohort 2). The population of people with CKD was defined as follows at index date: 18 years of age or older and availability of at least 12 months of database history. In addition, presence of CKD was based on either a recorded diagnosis of CKD (as ICPC code U99.01 and/or as a free-text description in the GP data) prior to index date and/or a CKD stage defined by estimated GFR (eGFR) in combination with urine albumin/creatinine ratio (UACR) based on Kidney Disease Improving Global Outcomes (KDIGO) criteria [11], a CKD stage defined by the GP, or CKD stage recorded as a primary discharge diagnosis in the hospital data.

The stage of CKD was determined where possible, using the two most recent eGFR measurements in the two years before the index date, with a minimum of 90 days between these measurements. The CKD stages were defined according to the KDIGO classification [11]. When the two measurements fell into different stages, the best stage of the most recent two measurements was used. When eGFR was missing, or only one eGFR was available, the stage established by the GP was used when available. If insufficient information was available in the GP data to classify the CKD stage, it was based on the primary discharge diagnosis in the hospital data. The stage was considered unspecified if the stage could not be determined based on the above criteria.

### Definitions of comorbidities

The diagnosis of type 1 or 2 diabetes mellitus (T1D and T2D) was determined step-by-step in the GP data:

1. People with a recorded diagnosis of T1D (ICPC code T90.01) before or on the day of the first prescription of short-acting insulin (ATC code A10AB) were classified as T1D;
2. People with a recorded diagnosis of T2D (ICPC code T90.02) within 12 months of the index date were classified as T2D;
3. People younger than 30 years of age with an insulin prescription (ATC code A10A) were classified as T1D;
4. People with a prescription of at least one blood glucose lowering therapy other than insulin (ATC code A10B) in the 6 months before the index date were classified as T2D;

People who did not meet the above criteria were considered unclassifiable and removed from the study. This concerned four patients.

A recorded diagnosis determined heart failure (ICPC code K77) and other comorbidities in the GP data before or on the index date. Cardiovascular disease at index date was defined as (a history of) ischemic heart disease, heart failure, atrial fibrillation, stroke and/or peripheral artery disease. Clinical parameters (UACR and systolic blood pressure) were determined based on the most recent value in the two years before the index date. Medication use was determined based on prescriptions in the year before the index date.

### Complications

The occurrence of all-cause mortality, cardiovascular mortality (defined as death during hospitalization with a cardiovascular diagnosis), renal mortality (defined as death during hospitalization with renal diagnosis), myocardial infarction, cerebrovascular events, hospitalization for heart failure, hospitalization for CKD, cardiorenal syndrome (defined as a concurrent diagnosis of both heart failure and CKD during hospitalization), decreased in eGFR values of at least 57% [12], kidney transplantation, and peripheral arterial vascular disease was recorded in the two years of follow-up. These complications were determined by primary discharge diagnosis as stated in the hospital data, except for a decrease in eGFR values, which was established using GP data. Per complication, the first event was used to determine the incidence rate. Complications were assessed in the entire study population with CKD in cohort 2 and in the subgroups of people with the additional diagnosis of T2D and/or heart failure.

### Statistics

Renal and cardiovascular complications are described as the incidence (incidence rate, (IR)) per 1,000 person-years. Since this is a descriptive study, no statistical hypothesis testing between groups was performed. A statistical analysis plan was written in advance for this study. The analyses were performed in SAS version 9.4.

### Data availability

The data that support the findings of this study are available from STIZON via the PHARMO Institute but restrictions apply to the availability of these data, which were used under license for the current study, and so are not publicly available. Data are however available from the authors upon reasonable request and with permission of STIZON.

## Results

### Prevalence of CKD

The prevalence of CKD in The Netherlands was 8.9% (n = 194,978) in an adult population of 2,187,962 people.

### Baseline characteristics and occurrence of Complications in people with CKD

**Fig. 1:**
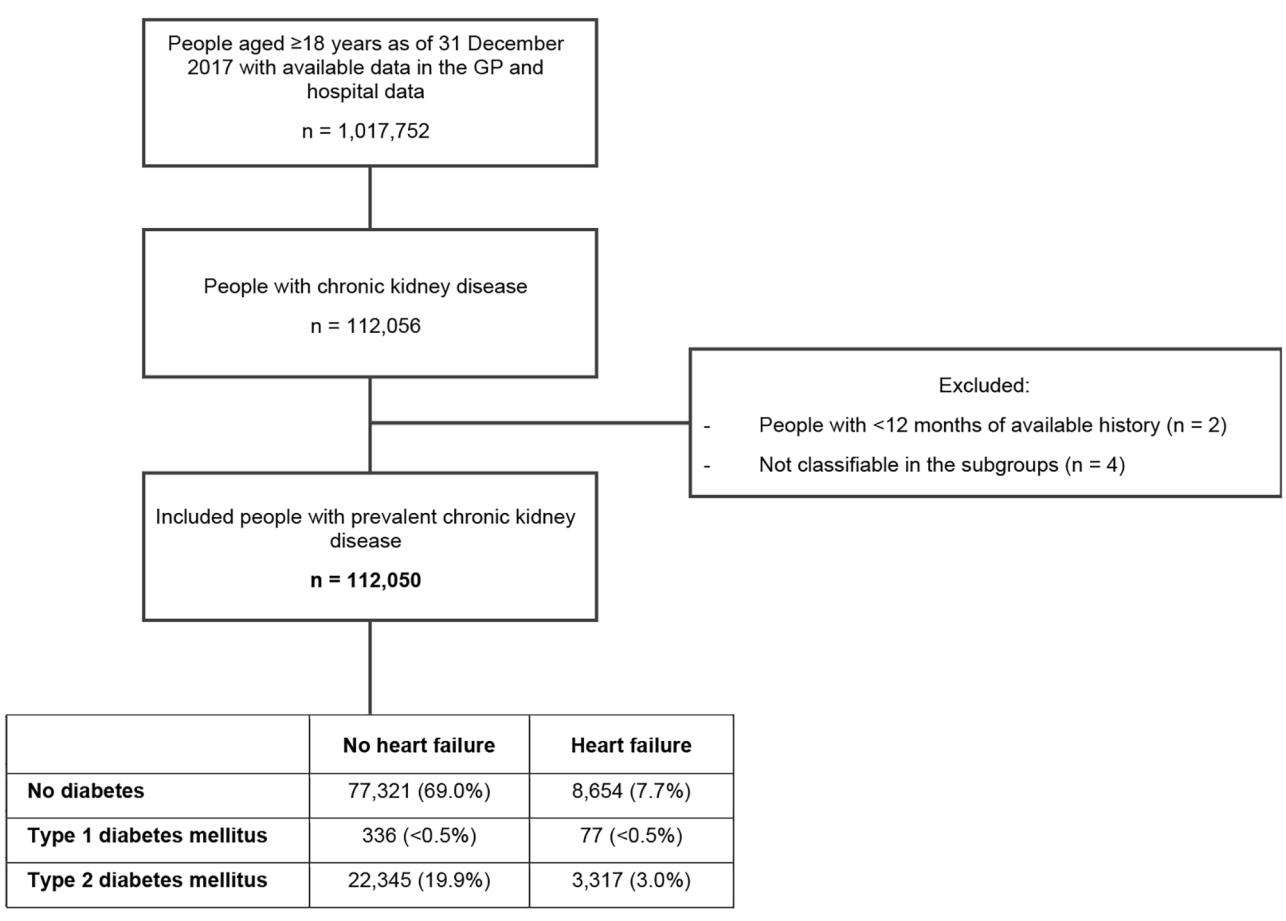
Study flowchart for people in the longitudinal analysis

For the analysis of renal and cardiovascular complications, 112,050 people with CKD were included who had GP, hospital, and outpatient pharmacy data available (Fig. 1). The mean (SD) age of this group was 72 [12] years, and 57% were females. The stage of CKD could be determined in 36% of people. Of the 40,451 people with a known stage, the majority had stage 3a (59%) or 3b (20%). The KDIGO risk categories, that also takes albuminuria into account [10] could be determined in approximately one third of the CKD patients. Of the patients with a known KDIGO risk category, 67% had a moderate risk, 21% a high risk and 12% a very high risk (Table 1 and Supplementary data; Figures S1-S5). Any cardiovascular disease co-existed in 63%, heart failure in 11%, and T2D in 23% (Table 1). Of the patients with a known KDIGO risk category, this was 68%, 12% and 31%, respectively (Supplementary data, Table S1). Given the small size of the T1D population (< 0.5%), this population was not further analyzed.

**Table 1:** Baseline characteristics

Parameters	Total population with CKD	CKD only	CKD + T2D	CKD + HF	CKD + T2D + HF
	N = 112,050	N = 77,321	N = 22,345	N = 8,654	N = 3,317
**Gender**					
Male	48,130 (43)	31,133 (40)	11,323 (51)	3,760 (43)	1,710 (52)
Female	63,920 (57)	46,188 (60)	11,022 (49)	4,894 (57)	1,607 (48)
**Age (years), mean (SD)**	72 (12)	72 (12)	71 (11)	81 (10)	77 (10)
**CKD stage**					
1	5,097 (5)	2,722 (4)	2,176 (10)	83 (1)	93 (3)
2	1,150 (1)	707 (1)	334 (1)	58 (1)	46 (1)
3a	23,826 (21)	16,671 (22)	4,279 (19)	2,001 (23)	837 (25)
3b	8,145 (7)	4,537 (6)	1,557 (7)	1,431 (17)	589 (18)
4	1,920 (2)	897 (1)	321 (1)	475 (5)	208 (6)
5	313 (< 0.5)	169 (< 0.5)	53 (< 0.5)	46 (1)	34 (1)
Not specified	71,599 (64)	51,618 (67)	13,625 (61)	4,560 (53)	1,510 (46)
**KDIGO risk categories [10]**					
Moderate risk	27,766 (25)	18,184 (24)	7,210 (32)	1,482 (17)	842 (25)
High risk	8,626 (8)	4,908 (6)	2,325 (10)	841 (10)	536 (16)
Very high-risk	5,079 (5)	2,359 (3)	1,280 (6)	878 (10)	534 (16)
Not specified	70,579 (63)	51,870 (67)	11,530 (52)	5,453 (63)	1,405 (42)
**Comorbidities**					
Cardiovascular disease	71,046 (63)	45,983 (59)	12,785 (57)	8,654 (100)	3,317 (100)
Ischemic heart disease	25,499 (23)	14,103 (18)	5,516 (25)	3,948 (46)	1,795 (54)
Heart failure	12,048 (11)	0	0	8,654 (100)	3,317 (100)
Atrial fibrillation	16,332 (15)	8,719 (11)	2,497 (11)	3,737 (43)	1,326 (40)
Cerebrovascular event	17,827 (16)	11,594 (15)	3,213 (14)	2,169 (25)	776 (23)
Peripheral artery disease	24,361 (22)	16,887 (22)	3,088 (14)	3,392 (39)	909 (27)
Diabetes mellitus					
Type 1 diabetes mellitus	413 (< 0.5%)				
Type 2 diabetes mellitus	25,662 (23%)				
Cancer	17,010 (15)	11,646 (15)	3,172 (14)	1,529 (18)	608 (18)
COPD	13,265 (12)	7,788 (10)	2,413 (11)	2,120 (24)	906 (27)
**Medication**					
ACE inhibitors	33,531 (30)	20,345 (26)	8,532 (38)	3,038 (35)	1,467 (44)
Angiotensin 2 inhibitors	29,039 (26)	18,783 (24)	6,759 (30)	2,306 (27)	1,054 (32)
Statins	57,979 (52)	34,631 (45)	16,554 (74)	4,142 (48)	2,397 (72)
**Clinical/laboratory parameters**					
UACR					
<3 mg/mmol	49,139 (44)	33,977 (44)	10,938 (49)	2,808 (32)	1,349 (41)
3–30 mg/mmol	22,794 (20)	13,692 (18)	6,618 (30)	1,452 (17)	971 (29)
>30 mg/mmol	2,868 (3)	1,393 (2)	1,010 (5)	213 (2)	234 (7)
Unknown	37,249 (33)	28,259 (37)	3,779 (17)	4,181 (48)	763 (23)
Systolic blood pressure (mmHg), mean (SD)	138 (17)	138 (17)	138 (16)	135 (19)	135 (19)

ACE, angiotensin converting enzyme; CKD, chronic kidney disease; COPD, chronic obstructive pulmonary disease; HF, heart failure; SD, standard deviation; T1D, type 1 diabetes mellitus; T2D, type 2 diabetes mellitus; UACR, urine albumin/creatinine ratio

Data presented as n (%), unless otherwise specified. Due to low numbers, the populations with CKD + T1D and CKD + T1D + heart failure are not included in this table

The most common first complications over 2 years of follow-up were cerebrovascular events (IR 11 per 1,000 person-years), hospitalizations for heart failure (IR 10 per 1,000 person-years), hospitalizations for CKD (IR 6.2 per 1,000 person-years) and myocardial infarction (IR 5.5 per 1,000 person-years). In 4.9 cases per 1,000 person-years, there was concomitant hospitalization for heart failure and CKD (cardiorenal syndrome). Mortality in the study population was 12 deaths per 1,000 person-years, of which 4.1 cases were cardiovascular, and 2.2 cases were renal deaths per 1,000 person-years.

**Fig. 2:**
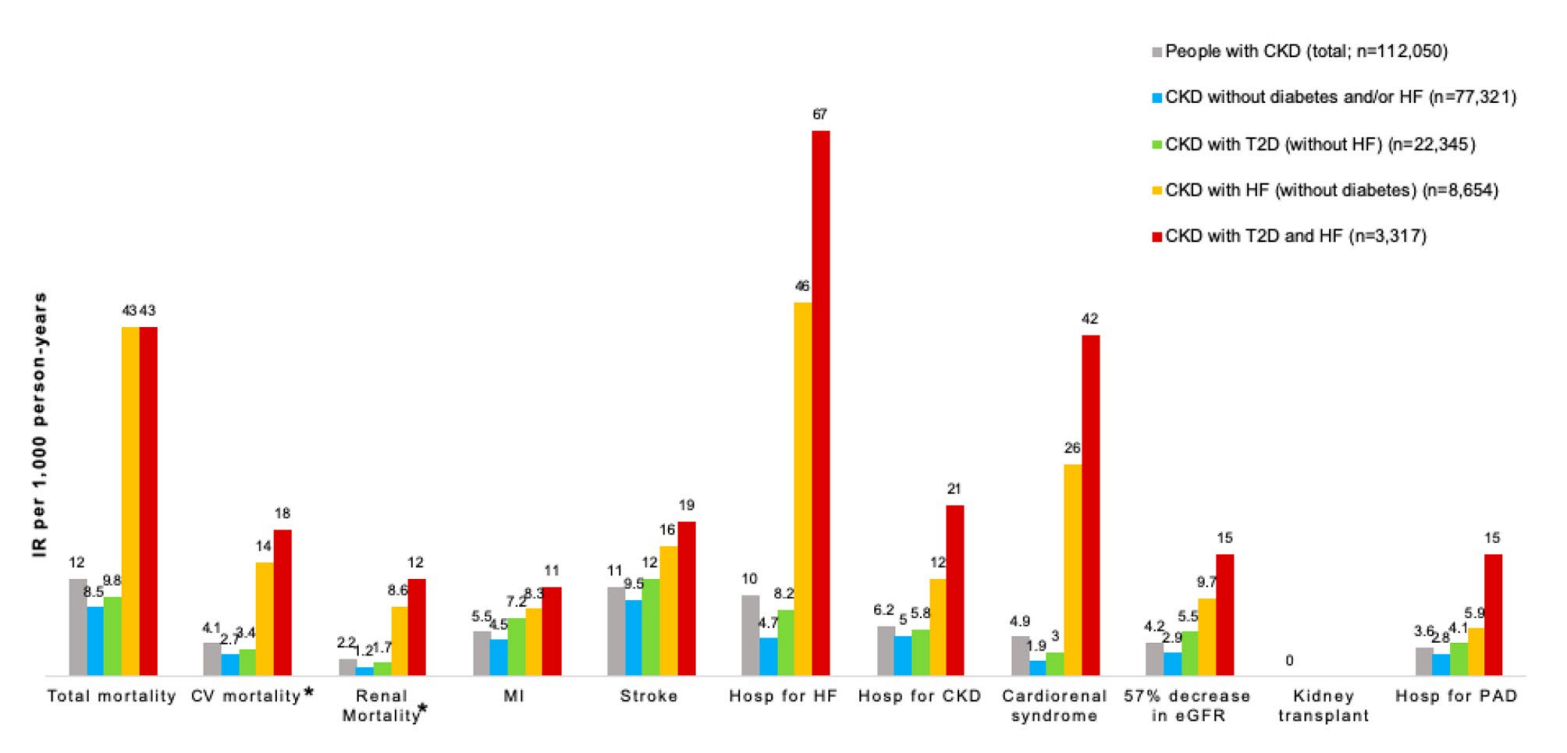
Complications of people with CKD over 2 years of follow-up. CKD, chronic kidney disease; CV, cardiovascular; eGFR, estimated glomerular filtration rate; HF, heart failure; Hosp, hospitalization; IR, incidence rate; MI, myocardial infarction; PAD, peripheral arterial disease; T2D, type 2 diabetes. *based on a proxy of cardiovascular and renal diagnoses, respectively, in case of death during hospitalization

The IRs for all complications studied, and mortality for people with CKD as well as T2D and/or heart failure are shown in Fig. 2 and Supplementary data; Table S2.

## Discussion

In this study, we found that the prevalence of established CKD was 8.9% in the adult population in The Netherlands on 31 December 2019.

The prevalence of CKD in this study was lower than the 10.4% reported in the PREVEND cohort, another study from our country [4]. PREVEND study differed from the current one in two fundamental aspects. First, in PREVEND, researchers actively screened for the presence of CKD, thereby detecting people who were not yet diagnosed with CKD, which would likely have led to a higher prevalence number. Second, people with albuminuria were overrepresented by how the cohort was composed, which would likely have overestimated the prevalence of CKD based on GFR criteria. The prevalence rate of CKD in our study was higher than in the study by Van Blijderveen et al., which reported a prevalence of 5.1% [5]. This study exclusively focused on data from primary care by general practitioners and used different criteria to define CKD. For example, people in whom CKD was diagnosed on a single eGFR estimate, followed by direct referral to the second-line, may not have been captured in the reported prevalence. Comparison of the prevalence of CKD in The Netherlands with other European countries is difficult, and a wide variety has been reported, ranging from 2.1 to 12.0% depending on the definition of CKD that was used [13, 14].

The presence of CKD is a well-established risk factor of substantial magnitude for cardiovascular complications and mortality [2, 10, 15]. In this study no comparison was made between the CKD population and a population without CKD. However, our data suggest that among people with CKD those who also have T2D and/or HF have a further increased risk for cardiovascular complications and mortality– even though this should be concluded with caution since no formal comparative analysis was performed in our study. Nevertheless, the presence of these co-morbidities besides CKD may identify individuals with the highest risk. Of note, these people are also under the care of general practitioners in The Netherlands. Recognizing such subgroups can provide health benefits if cardiovascular risk management could be further optimized in either primary or hospital-based care. For people with CKD, this includes striving for blood pressure below 130/80 mmHg [9]. In the cohort we investigated, the systolic blood pressure was 138 ± 17 mmHg. Although we were unable to investigate how blood pressure was measured and whether contraindications existed to pursue this treatment goal, our data suggest that improvements are possible. The same applies to renin-angiotensin-aldosterone system (RAAS) inhibitors, which were used in only 54% of people, possibly pointing to a window of opportunity for improvement. However, it is important to note that in our analysis only the medication that was prescribed by the GP (including prescriptions continued from initial prescriptions by a medical specialist) was available.

This study has some limitations. The CKD stage was unknown in 64% of our population, Since it is conceivable that among people with comorbidity more extensive staging will be performed, the prevalence of comorbity may differ depending on knowledge or not of KDIGO risk category. Supplemental Table 2 shows this is the case. In addition, while for those where the stage could be determined on reported eGFR values, the used formula could not be retrieved from the data sources, and could have been the ‘modification of diet in renal disease’ (MDRD) or the CKD-epi formula. However, the bias between the two formulas in the eGFR range of our study cohort is small, and therefore we assume that the chance of misclassification is small as well. Also, data from clinical records were used, and no screening in asymptomatic persons was performed except for people with T2D, where Dutch guidelines advocate active screening for the presence of CKD, including screening for albuminuria. In the persons without diabetes, there most likely has been a clinical incentive to investigate the presence of kidney disease. Therefore, it is possible that our analysis underestimates the true prevalence of CKD.

Moreover, because the recorded diagnosis has been used for a proportion of people with CKD, instead of laboratory-based criteria to define CKD, it is possible that misclassification may have led to either under- or overreporting of prevalence data and the impact of CKD on events. In addition, the GP may have registered CKD as such, but not the stage of CKD. Therefore, the differential impact of varying stages of CKD on the complication risks could not be studied. However, our research question was not to assess this impact since this is well-established in the existing literature. Instead, in our study, besides on the prevalence of CKD, we focused on the cardiovascular and renal complications of CKD and described the added risk of comorbidity on top of CKD. A final generic limitation on studies using registries is that when the presence of CKD is based on a recorded diagnosis, it cannot be excluded that the actual ciagnosis may have been Acute Kidney Injury, even if IPCP cosing system does separate the two. In our analysis, the first complication was used in the analysis. However, non-fatal medical complications may recur; therefore, the impact of CKD with or without comorbidities is likely higher than we reported. Moreover, our follow-up period was relatively short. Finally, our data are observational, and we did not apply statistical testing.

Our study also has several strengths. First, this is a study of the largest Dutch population to date in a database representative of the Dutch population [8]. The data used are more recent than those from previous analyses of the Dutch situation, as mentioned above. Moreover, here we linked data relating to first-line, second-line, and pharmacist healthcare data, thereby capturing a wide range of relevant data for this analysis. This also optimized the registration of complications. Finally, our analysis is the first to examine the impact of the co-existence of heart failure and T2D, in addition to CKD, on the development of complications. It must be noted that an independent causal relationship between these comorbidities and the outcomes cannot be concluded from our study since we did not statistically test this for potential confounding. However, our research aimed not to test for this independent causal relationship.

## Conclusions

In conclusion, the prevalence rate of established CKD in The Netherlands is 8.9%. People with CKD and T2D, heart failure, or both had numerically higher mortality and cardiorenal complication rates than those without these comorbidities. Implementation of up-to-date cardiovascular risk management in this group of people could provide improved health benefits.

### Electronic supplementary material

Below is the link to the electronic supplementary material.

Supplementary Material 1

## Data Availability

Requests for sharing study data must be made on specific grounds either with the aim to corroborate the study results in the interest of Public Health or in the context of an audit by a competent authority. Such requests should be made to corresponding author, who will forward the request to JO. Sufficient information needs to be provided to confirm that the request is made for one of the above-mentioned purposes, including a wound justification and, in case of a request with a view to corroborate study results, a protocol on the research for which the data will be used or a plan for quality control checks, as applicable.

## Abbreviations

ACE: angiotensin converting enzyme
CI: confidence interval
CKD: chronic kidney disease
COPD: chronic obstructive pulmonary disease
COVID: coronavirus disease
CV: cardiovascular
eGFR: estimated glomerular filtration rate
ESKD: end-stage kidney disease
GP: general practitioner
GFR: glomerular filtration rate
HF: heart failure
Hosp: hospitalization
IR: incidence rate
ICPC: International Classification of Primary Care
KDIGO: Kidney Disease Improving Global Outcomes
MDRD: modification of diet in renal disease
MI: myocardial infarction
NZa: (*Nederlandse Zorgautoriteit* [Dutch Healthcare Authority
PAD: peripheral arterial disease
PY: person-years
RAAS: renin-angiotensin-aldosterone system
SD: standard deviation
T2D: type 2 diabetes mellitus
T1D: type 1 diabetes mellitus
UACR: urine albumin/creatinine ratio
