# Supplementary material for: Prevalence of chronic kidney disease in the Netherlands and its cardiovascular and renal complications
Source: BMC Nephrol. 2023 Nov 13;24:337. doi: 10.1186/s12882-023-03384-y (PMC10644456; doi:10.1186/s12882-023-03384-y)
Supplement: Supplementary file 1 — Supplementary Material 1 [file 12882_2023_3384_MOESM1_ESM.docx]

**SUPPLEMENTARY TABLE AND FIGURES**

**Table S1. Baseline characteristics of population with known KDIGO category (i.e. known eGFR and UACR)**

| **Parameters** | **Total population with CKD** | **CKD only** | **CKD + T2D** | **CKD + HF** | **CKD + T2D + HF** |
| --- | --- | --- | --- | --- | --- |
|  | **N = 40,871** | **N = 25,174** | **N = 10,759** | **N = 2,984** | **N = 1,869** |
| **Gender** |  |  |  |  |  |
| Male | 18,681 (46) | 10,737 (43) | 5,624 (52) | 1,318 (44) | 956 (51) |
| Female | 22,190 (54) | 14,437 (57) | 5,135 (48) | 1,666 (56) | 913 (49) |
| **Age (years), mean (SD)** | 74 (11) | 74 (11) | 72 (11) | 81 (8) | 78 (9) |
| **CKD stage** |  |  |  |  |  |
| 1 | 4,287 (10) | 2,253 (9) | 1,863 (17) | 72 (2) | 78 (4) |
| 2 | 795 (2) | 432 (2) | 283 (3) | 37 (1) | 39 (2) |
| 3a | 16,465 (40) | 10,953 (44) | 3,543 (33) | 1,247 (42) | 700 (37) |
| 3b | 5,637 (14) | 3,062 (12) | 1,296 (12) | 800 (27) | 467 (25) |
| 4 | 1,088 (3) | 482 (2) | 236 (2) | 213 (7) | 150 (8) |
| 5 | 69 (<0.5) | 31 (<0.5) | 13 (<0.5) | 14 (<0.5) | 10 (1) |
| Not specified | 12,530 (31) | 7,961 (32) | 3,525 (33) | 601 (20) | 425 (23) |
| **KDIGO risk categories [10]** |  |  |  |  |  |
| Moderate risk | 27,766 (68) | 18,184 (72) | 7,210 (67) | 1,482 (50) | 842 (45) |
| High risk | 8,626 (21) | 4,908 (19) | 2,325 (22) | 841 (28) | 536 (29) |
| Very high-risk | 4,479 (11) | 2,082 (8) | 1,224 (11) | 661 (22) | 491 (26) |
| Not specified | NA | NA | NA | NA | NA |
| **Comorbidities** |  |  |  |  |  |
| Cardiovascular disease | 27,765 (68) | 16,171 (64) | 6,683 (62) | 2,984 (100) | 1,869 (100) |
| Ischemic heart disease | 10,173 (25) | 4,961 (20) | 2,857 (27) | 1,301 (44) | 1,025 (55) |
| Heart failure | 4,874 (12) | - | - | 2,984 (100) | 1,869 (100) |
| Atrial fibrillation | 6,503 (16) | 3,060 (12) | 1,370 (13) | 1,285 (43) | 777 (42) |
| Cerebrovascular event | 7,320 (18) | 4,397 (17) | 1,676 (16) | 782 (26) | 446 (24) |
| Peripheral artery disease | 10,444 (26) | 6,907 (27) | 1,629 (15) | 1,368 (46) | 518 (28) |
| Diabetes mellitus |  |  |  |  |  |
| Type 1 diabetes mellitus | 85 (<0.5) | - | - | - | - |
| Type 2 diabetes mellitus | 12,628 (31) | - | 10,759 (100) | - | 1,869 (100) |
| Cancer | 6,260 (15) | 3,807 (15) | 1,587 (15) | 514 (17) | 345 (18) |
| COPD | 5,140 (13) | 2,694 (11) | 1,242 (12) | 729 (24) | 467 (25) |
| **Medication** |  |  |  |  |  |
| ACE inhibitors | 14,320 (35) | 7,956 (32) | 4,396 (41) | 1,081 (36) | 851 (46) |
| Angiotensin 2 inhibitors | 11,976 (29) | 7,051 (28) | 3,426 (32) | 856 (29) | 612 (33) |
| Statins | 24,245 (59) | 13,239 (53) | 8,016 (75) | 1,552 (52) | 1,381 (74) |
| **Clinical/laboratory parameters** |  |  |  |  |  |
| UACR |  |  |  |  |  |
| <3 mg/mmol | 20,008 (49) | 13,345 (53) | 4,277 (40) | 1,584 (53) | 780 (42) |
| 3-30 mg/mmol | 17,439 (43) | 10,038 (40) | 5,366 (50) | 1,152 (39) | 836 (45) |
| >30 mg/mmol | 2,189 (5) | 972 (4) | 833 (8) | 168 (6) | 202 (11) |
| Unknown | 1,235 (3) | 819 (3) | 283 (3) | 80 (3) | 51 (3) |
| Systolic blood pressure (mmHg), mean (SD) | 138 (17) | 138 (17) | 139 (17) | 135 (18) | 135 (18) |
| ACE, angiotensin converting enzyme; CKD, chronic kidney disease; COPD, chronic obstructive pulmonary disease; HF, heart failure; SD, standard deviation; T1D, type 1 diabetes mellitus; T2D, type 2 diabetes mellitus; UACR, urine albumin/creatinine ratio.  Data presented as n (%), unless otherwise specified. Due to low numbers, the populations with CKD + T1D and CKD + T1D + heart failure are not included in this table. | | | | | |

**Table S2.** Complications of people with CKD over 2 years of follow-up

|  | **Total patients with CKD** | | | **CKD without DM and HF** | | | **CKD with T2D (without HF)** | | | **CKD with HF (without DM)** | | | **CKD with T2D and HF** | | |
| --- | --- | --- | --- | --- | --- | --- | --- | --- | --- | --- | --- | --- | --- | --- | --- |
|  | **Nat risk = 112,050** | | | **Nat risk = 77,321** | | | **Nat risk = 22,345** | | | **Nat risk = 8,654** | | | **Nat risk = 3,317** | | |
|  | n  events | PY | IR/1,000 PY  (95% CI) | n  events | PY | IR/1,000 PY  (95% CI) | n  events | PY | IR/1,000 PY  (95% CI) | n  events | PY | IR/1,000 PY  (95% CI) | n  events | PY | **IR / 1,000 PY**  (95% CI) |
| Total mortality | 2,192 | 179,956 | **12 (12-13)** | 1,064 | 125,047 | **8.5 (8.0-9.0)** | 358 | 36,612 | **9.8 (8.8-**  **10.8)** | 550 | 12,724 | **43 (40-47)** | 210 | 4,909 | **43 (37-49)** |
| Cardiovascular mortality^a^ | 733 | 179,956 | **4.1 (3.8-4.4)** | 333 | 125,047 | **2.7 (2.4-3.0)** | 124 | 36,612 | **3.4 (2.8-4.0)** | 182 | 12,724 | **14 (12-17)** | 90 | 4,909 | **18 (15-22)** |
| Renal mortality^a^ | 388 | 179,956 | **2.2 (1.9-2.4)** | 154 | 125,047 | **1.2 (1.0-1.4)** | 61 | 36,612 | **1.7 (1.3-2.1)** | 109 | 12,724 | **8.6 (7.0-10.3)** | 61 | 4,909 | **12 (9-16)** |
| Myocardial infarction | 987 | 179,097 | **5.5 (5.2-5.9)** | 555 | 124,556 | **4.5 (4.1-4.8)** | 262 | 36,383 | **7.2 (6.3-8.1)** | 105 | 12,639 | **8.3 (6.8-10.0)** | 55 | 4,862 | **11 (8-15)** |
| Cerebrovascular event | 1,924 | 178,445 | **11 (10-11)** | 1,183 | 124,098 | **9.5 (9.0-10.1)** | 444 | 36,253 | **12 (11-13)** | 198 | 12,586 | **16 (14-18)** | 92 | 4,848 | **19 (15-23)** |
| Hospitalization for HF | 1,781 | 178,705 | **10 (9.5-10.4)** | 590 | 124,643 | **4.7 (4.4-5.1)** | 297 | 36,385 | **8.2 (7.2-9.1)** | 573 | 12,334 | **46 (43-50)** | 313 | 4,685 | **67 (59-75)** |
| Hospitalization for CKD | 1,101 | 178,973 | **6.2 (5.8-6.5)** | 623 | 124,487 | **5.0 (4.6-5.4)** | 211 | 36,424 | **5.8 (5.0-6.6)** | 151 | 12,592 | **12 (10-14)** | 99 | 4,825 | **21 (17-25)** |
| Cardiorenal syndrome | 874 | 179,405 | **4.9 (4.5-5.2)** | 236 | 124,907 | **1.9 (1.7-2.1)** | 109 | 36,544 | **3.0 (2.4-3.6)** | 322 | 12,518 | **26 (23-29)** | 199 | 4,778 | **42 (36-48)** |
| 57% decrease in eGFR | 762 | 179,425 | **4.2 (3.9-4.6)** | 361 | 124,750 | **2.9 (2.6-3.2)** | 202 | 36,485 | **5.5 (4.8-6.3)** | 123 | 12,661 | **9.7 (8.0-11.5)** | 71 | 4,868 | **15 (11-18)** |
| Kidney transplant | 2 | 179,954 | **0.01 (0-0.03)** | 0 | 125,047 | **-** | 0 | 36,612 | **-** | 0 | 12,724 | **-** | 0 | 4,909 | **-** |
| Hospitalization for  peripheral arterial vascular disease | 648 | 179,366 | **3.6 (3.3-3.9)** | 344 | 124,724 | **2.8 (2.5-3.1)** | 148 | 36,473 | **4.1 (3.4-4.8)** | 75 | 12,664 | **5.9 (4.6-7.4)** | 71 | 4,849 | **15 (11-18)** |
| CI, confidence interval; CKD, chronic kidney disease; DM, diabetes mellitus; eGFR, estimated glomerular filtration rate; HF, heart failure; IR, incidence rate; PY, person-years; T2D, type 2 DM.  ^a^based on a proxy of cardiovascular and renal diagnoses, respectively in case of death during hospitalization. | | | | | | | | | | | | | | | |

**Figure S1.** Number of patients per KDIGO risk category - Total CKD population

*Although UACR was unknown, these classify as ‘very high risk’ in this eGFR range

**Figure S2.** Number of patients per KDIGO risk category – CKD only

*Although UACR was unknown, these classify as ‘very high risk’ in this eGFR range

**Figure S3.** Number of patients per KDIGO risk category – CKD + T2D

*Although UACR was unknown, these classify as ‘very high risk’ in this eGFR range

**Figure S4.** Number of patients per KDIGO risk category – CKD + HF

*Although UACR was unknown, these classify as ‘very high risk’ in this eGFR range

**Figure S5.** Number of patients per KDIGO risk category – CKD + T2D + HF

*Although UACR was unknown, these classify as ‘very high risk’ in this eGFR range
